# Paradigm Shift toward Reducing Overtreatment of Ductal Carcinoma *In Situ* of Breast

**DOI:** 10.3389/fonc.2017.00192

**Published:** 2017-08-28

**Authors:** Yasuaki Sagara, Wong Julia, Mehra Golshan, Masakazu Toi

**Affiliations:** ^1^Breast Cancer Unit, Kyoto University Hospital Breast Surgery, Graduate School of Medicine, Kyoto University, Kyoto, Japan; ^2^Department of Breast Surgical Oncology, Hakuaikai Social Medical Cooperation, Kagoshima, Japan; ^3^Department of Surgery, Brigham and Women’s Hospital, Boston, MA, United States; ^4^Department of Radiation Oncology, Dana-Farber Cancer Institute, Boston, MA, United States

**Keywords:** ductal carcinoma *in situ*, surgery, radiotherapy, hormonal therapy, adjuvant therapy

## Abstract

The prevalence of ductal carcinoma *in situ* (DCIS) of the breast has increased substantially after the introduction of breast cancer screening programs, although the clinical effects of early DCIS detection and treatment remain unclear. The standard treatment for DCIS has involved local breast-conserving surgery (BCS) followed by radiotherapy (RT) or total mastectomy with/without endocrine therapy, and the choice of local treatment is not usually based on clinicopathologic or biological factors. However, we have investigated the effectiveness of local treatment using breast surgery and RT using Surveillance, Epidemiology, and End Results data, and found that the effectiveness of breast surgery was modified by the nuclear grade. Furthermore, breast cancer-specific survival was identical between patients with low-grade DCIS who did and did not undergo surgery. Moreover, we found that RT after BCS for DCIS was only associated with a survival benefit among patients with risk factors for local recurrence, such as nuclear grade, age, and tumor size. Ongoing clinical trials and translational research have attempted to develop a treatment strategy that prevents the overdiagnosis and overtreatment of low-risk DCIS, as well as a biology-based treatment strategy for using targeted therapy. Therefore, to develop a tailored treatment strategy for DCIS, we need to identify molecular and biological classifications based on the results from translational research, national databases, and clinical trials.

## Introduction of Breast Screening Programs and the Increased Prevalence of Ductal Carcinoma *In Situ* (DCIS)

Ductal carcinoma *in situ* of the breast is an abnormal proliferation of epithelial cells within the breast ducts. The implementation of breast screening programs from the 1980s helped improve the prognosis of women who were diagnosed with breast cancer, although the incidences of DCIS, early breast cancer, and slow-growing breast cancer have also increased dramatically (1–4). For example, the incidence of DCIS increased from 1.87 cases per 100,000 population during the 1970s to 32.5 cases per 100,000 population during 2004, and the incidence has currently reached a plateau (5, 6). A systematic review of the incidences before and after the implementation of breast cancer screening programs in five countries also revealed a dramatic increase in the incidence of breast cancer (7). The incidence in the United Kingdom increased approximately 40% above the estimated natural increase. Thus, if early-stage breast cancers are detected during breast cancer screening, it would be logical to assume that the incidence of advanced breast cancer should decrease. However, there has been no relative decrease in the incidence of advanced breast cancer, which suggests that there is overdiagnosis of slow-growing lesions that may not require treatment (1, 8–10). Although 3–40% of the lesions detected by screening are assumed to be overdiagnosis in several studies, we have not confirmed it yet and this is an area of some uncertainty (11, 12).

## Pathological Diagnosis and Molecular Classification of DCIS

Several retrospective studies have investigated the natural course of DCIS in the absence of curative treatment, and found progression to invasive breast cancer in 25–50% of cases during follow-ups of 15–25 years (13, 14). Thus, it is important to be aware that not all DCIS will become invasive breast cancer that can metastasize to other organs. Furthermore, a study of 6,900 slides from breast biopsies revealed variability in the pathological diagnoses (15), with 69.6% of pathologists providing diagnoses of DCIS and 18.5% of pathologists providing diagnoses of benign tissue or atypia. Therefore, as there is a broad biological spectrum of breast lesions that ranges from benign to invasive ductal carcinoma, it is important for physicians to consider the diagnostic gray area in clinical decision-making.

Silverstein et al. have demonstrated that the Van Nuys score based on nuclear grade and comedo necrosis is associated with local recurrence after breast-conserving surgery (BCS) for DCIS. This score is relatively reproducible to be implemented in clinical practice (16). The fourth edition of the World Health Organization’s Classification of Tumors of the Breast (2012) classified tumors based on nuclear grade, and DCIS has come to be classified as a low-grade, intermediate-grade, or high-grade lesion. Ozanne et al. have estimated that the cumulative rates of progression from DCIS to invasive cancer during a 10-year period are 60% for high-grade DCIS (patients who are <45 years old and have lesions that are >1 cm) and 16% for low-grade DCIS (patients who are >45 years old and have lesions that are >2.5 cm) (17). After local therapy for DCIS, nuclear grade has been shown to predict ipsilateral breast cancer recurrence in a randomized clinical trial and meta-analysis (18–20). Furthermore, comprehensive investigation of DCIS gene expressions revealed that low-grade DCIS and atypical ductal hyperplasia share a common chromosomal abnormality, while high-grade DCIS exhibits molecular profiles that are indistinguishable from invasive breast cancer (21, 22). Changing the terminology for low-grade DCIS currently referred to as “carcinoma” will allow physicians to shift medicolegal notions and perceived risk to reflect the evolving understanding of biology (3). Although several studies have attempted to create a molecular classification of DCIS cases, the gene expression profile for predicting progression to invasive breast cancer has not been clarified (23, 24). Therefore, we also need to develop a clinically useful classification system or a new treatment strategy for the lesions that are diagnosed as DCIS.

## Surgery for DCIS Based on the Biology

**Figure 1:**
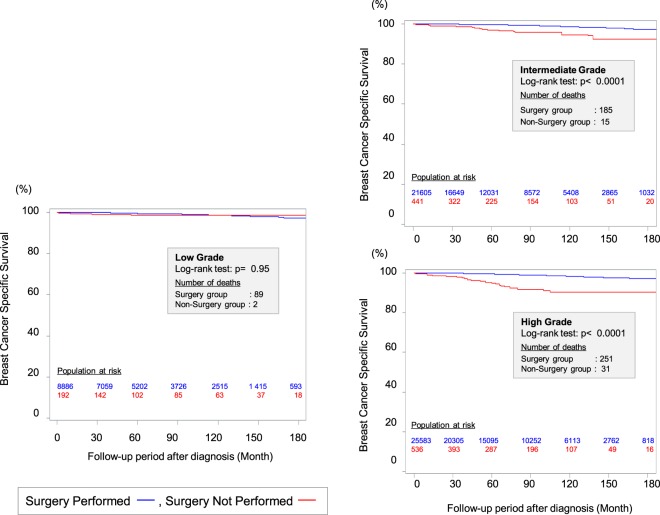
Kaplan–Meier curves for breast cancer-specific survival between surgery group and non-surgery group among patients weighted by inverse propensity score. Sagara et al. (25)

The standard local therapy for DCIS is BCS followed by radiotherapy (RT) or total mastectomy. However, local treatments have not usually been individualized based on the likelihood of progression to invasive breast cancer and distant metastasis. Therefore, we performed a retrospective cohort study to investigate the effectiveness of surgery for DCIS based on nuclear grade using Surveillance, Epidemiology, and End Results data (25). We used a method of propensity score weighting to adjust covariates that influence the prognosis of patients between surgery and non-surgery groups. Among the 57,222 eligible women with DCIS and a pathologically confirmed nuclear grade, we identified 1,169 women who did not undergo surgery for DCIS at the diagnosis. This decision was motivated by their physician not recommending surgery (46.8%), their physician not recommending surgery because of contraindications (1.7%), patient refusal despite a physician’s recommendation (9.8%), and unknown reasons despite a physician’s recommendation (40.9%). We observed a better breast cancer-specific survival (BCSS) among patients who underwent surgery for high-grade DCIS, compared to patients with high-grade DCIS who did not undergo surgery at a median follow-up of 72 months from diagnosis. However, the BCSS rates were identical for patients with low-grade DCIS who did and did not undergo surgery (Figure 1). Among patients with low-grade DCIS, the weighted 10-year BCSS rates were 98.6% after surgery and 98.8% among patients who did not undergo surgery. Thus, it may be prudent to reconsider the necessity of surgical treatment after a diagnosis of low-grade DCIS. Several randomized controlled trials (RCTs), such as the COMET (NCT02926911) and LORIS trials, are currently investigating the feasibility and non-inferiority of active surveillance with or without endocrine therapy for managing low-risk DCIS (26).

## RT for DCIS Based on Risk Factors and Gene Expressions

A meta-analysis of four RCTs (*n* = 3,729) revealed that RT after BCS for DCIS provided a decreased risk of local recurrence (hazard ratio: 0.46), although RT did not improve the BCSS (27). However, the limited number of deaths caused by breast cancer (*n* = 96) may have limited the power of the survival analysis. Interestingly, the benefit of RT (reducing local recurrence without a survival benefit) is balanced by several drawbacks, including adverse events, cost, and a prolonged treatment period. Therefore, there is substantial physician- and center-specific variability in the decision to perform or omit RT after BCS for DCIS.

**Figure 2:**
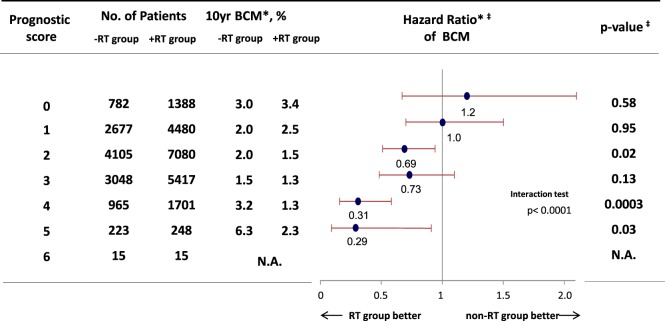
Hazard ratio comparing BCM among patients who received breast-conserving surgery for ductal carcinoma *in situ* between RT group and non-RT group. *Weighted by inverse propensity score. ^‡^Multivariate analysis adjusted by age of patients, year of diagnosis, race, tumor size, nuclear grade, and marital status. Abbreviation: RT, radiotherapy; BCM, breast cancer mortality. Sagara et al. (28).

We hypothesized that RT would provide a survival benefit to patients with risk factors for local recurrence, such as young age, large tumor size, and higher nuclear grade. Thus, we performed a cohort study using Surveillance, Epidemiology, and End Results data (1988–2007), and evaluated the efficacy of RT among 32,144 eligible women with DCIS and pathological data regarding tumor size and nuclear grade (28). We used the prognostic score that was proposed by Smith et al. to stratify the DCIS cases according to their risk of recurrence (29), which is associated with patient age, tumor size, and nuclear grade (higher scores are associated with local recurrence). The results confirmed that the prognostic score predicted both local recurrence and the survival benefit from RT among patients with the risk factors for local recurrence (Figure 2). Thus, the prognostic score could predict the risk of local recurrence and possible benefit of RT among patients who undergo BCS for DCIS.

The Oncotype Dx DCIS assay evaluates 12 genes to predict the risk of local recurrence after BCS for DCIS and has been validated in the Eastern Cooperative Oncology Group (ECOG) 5194 study and a study of a population-based cancer registry (30, 31). In the ECOG study, about 30% of the patients received tamoxifen. The DCIS score independently predicted the risk of recurrence after only BCS for DCIS, with 10-year ipsilateral breast event rates of 10.6% in the low-risk group, 26.7% in the intermediate-risk group, and 25.9% in the high-risk group (log rank *p* = 0.006). In a previous report from the ECOG study, the 7-year ipsilateral breast event rate was 10.5% for low- or intermediate-grade DCIS (32). Thus, the ipsilateral breast event rates appear to be similar between the low-risk group based on the DCIS score and low-grade DCIS cases. Modern clinical study of Radiation Therapy Oncology Group 9804 showed that BCS with RT have single digit local recurrence rates of only 0.9% at median 7.2-years follow-up period (33). Further studies are needed to confirm the incremental benefit that is provided by examining the patient’s genetic profile, compared to the classic clinicopathologic factors.

## Role of Systematic Therapy for DCIS

Adjuvant endocrine therapy can reduce the risks of ipsilateral recurrence and contralateral breast cancer after BCS for hormone receptor-positive DCIS. Meta-analysis of two RCTs revealed that the risks of ipsilateral and contralateral breast cancers were decreased by approximately 50% after BCS followed by adjuvant tamoxifen for DCIS. The absolute 10-year reduction was 6.5% for all new breast events after tamoxifen treatment, and the number needed to treat was 15 for preventing one recurrent event (34, 35). Based on these results, the use of endocrine therapy has increased steadily (36, 37). A large RCT (IBIS-II) has recently compared the risks of local recurrence after BCS for DCIS between groups that received an antiestrogen agent (tamoxifen) or an aromatase inhibitor (anastrozole) (38). As both treatments provided similar efficacies, a 5-year adjuvant treatment using tamoxifen is still considered the standard endocrine therapy for DCIS after BCS.

Several studies are currently evaluating the efficacies of systemic therapies that target the underlying biology of DCIS. For example, the Cancer and Leukemia Group B 40903 trial is evaluating neoadjuvant endocrine therapy using letrozole for hormone receptor-positive DCIS (NCT01439711). That study may provide further information regarding the mechanism of endocrine therapy and suitable biomarkers. As retrospective studies have demonstrated that recurrence is more common in human epidermal growth factor receptor 2 (HER2)-positive DCIS, compared to other DCIS subtypes (39, 40), the use of anti-HER2 therapy has been suggested for HER2-positive DCIS. A small prospective phase II single-arm study has also demonstrated that lapatinib (a tyrosine kinase inhibitor) interrupts the HER2/neu and epidermal growth factor receptor pathways, and significantly decreases the expressions of pHER2 and pERK1 in patients with HER2-positive DCIS. However, it did not alter the expression of Ki-67 (a proliferation marker) (41). The National Surgical Adjuvant Breast Project B-43 trial is a large phase III RCT (target recruitment: 2,000 patients) that is evaluating the addition of trastuzumab to standard treatment using surgery and RT (NCT00769379) (42). However, the benefit of adding targeted therapy for disease with favorable prognosis should outweigh the high costs (43).

## Conclusion and Perspective

Previous epidemiological studies have highlighted the issue of overdiagnosis and overtreatment of lesions that are detected during breast cancer screening. Furthermore, clinicians are confronted by a broad histological spectrum that ranges from normal tissue to invasive ductal carcinoma. Therefore, the ongoing clinical trials are needed to clarify the optimal management of DCIS. One strategy is active surveillance for low-risk DCIS, which is unlikely to develop into life-threatening disease, and systemic therapy may also be used to target the underlying biology of DCIS. Based on the steady increase in the use of adjuvant therapy, clinicopathologic factors and molecular profiles are needed to guide treatment based on the possibility of progression to invasive ductal carcinoma. Nevertheless, it may be prudent to de-escalate the comprehensive treatment for DCIS in select cases based on the tumor’s biology, which may reduce the overdiagnosis and overtreatment of lesions that are detected by breast cancer screening programs. While we wait for better molecular or other tests to risk-stratify DCIS, clinical decisions with the patients must be guided by information regarding the treatment’s benefits (reduced risk of local recurrence and increased survival) and drawbacks (e.g., comorbidities and cost).

## Author Contributions

All the authors contributed to manuscript writing and final approval of manuscript.

## Conflict of Interest Statement

The authors declare that the research was conducted in the absence of any commercial or financial relationships that could be construed as a potential conflict of interest.
